# Subscribers’ perception of quality of services provided by Ghana’s National Health Insurance Scheme - what are the correlates?

**DOI:** 10.1186/s12913-019-4023-3

**Published:** 2019-03-28

**Authors:** Edward Nketiah-Amponsah, Robert Kaba Alhassan, Samuel Ampaw, Aaron Abuosi

**Affiliations:** 1 Department of Economics, University of Ghana, Legon, Accra Ghana; 2 School of Nursing and Midwifery, University of Health and Allied Sciences, Ho, Ghana; 3 Department of Public Administration and Health Services Management, University of Ghana, University of Ghana Business School, Legon, Accra Ghana

**Keywords:** Quality care, National Health Insurance Scheme, Rural-urban, Ordered logistics regression, Demographic and health survey, Ghana

## Abstract

**Background:**

Ghana’s National Health Insurance Scheme (NHIS) has witnessed an upsurge in enrollment since its inception in 2003, with over 40% of the Ghanaian population actively enrolled in the scheme. While the scheme strives to achieve universal health coverage, this quest is derailed by negative perceptions of the quality of services rendered to NHIS subscribers. This paper presents an analysis on perceptions of service quality provided to subscribers of Ghana’s NHIS with emphasis on rural and urban scheme policy holders, using a nationally representative data.

**Methods:**

The study used data from the 2014 Ghana Demographic and Health Survey. Ordered logistic regressions were estimated to identify the correlates of perceived quality of care of services rendered by the NHIS. Also, chi-square statistics were performed to test for significant differences in the proportions of subscribers in the two subsamples (rural and urban).

**Results:**

Rural subscribers of the NHIS were found to identify more with better perception of quality of services provided by the NHIS than urban subscribers. Results from the chi-square statistics further indicated that rural subscribers are significantly different from urban subscribers in terms of the selected socioeconomic and demographic characteristics. In the full sample; age, out-of-pocket payment for healthcare and region of residence proved significant in explaining perceived quality of services rendered by the NHIS. Age, out-of-pocket payment for healthcare, region of residence, wealth status, and access to media were found to be significant predictors of perceived quality of services provided to both rural and urban subscribers of the NHIS. The significance of these variables varied among men and women in rural and urban areas.

**Conclusion:**

Different factors affect the perception of quality of services provided to rural and urban subscribers of Ghana’s NHIS. Health financing policies geared toward improving the NHIS-related services in rural and urban areas should be varied.

## Background

The government of Ghana has over the past one and a half decades intensified its quest to achieve universal health coverage. Consequently, Ghana implemented a national health insurance scheme (NHIS) in 2005 as the first of its kind in sub-Saharan Africa. The scheme obligates all Ghanaian citizens and residents of Ghana to subscribe to a health insurance scheme as enshrined in the NHIS amended Act 852 (2012) [1]. Nevertheless, less than 50% of the Ghanaian population are currently enrolled due largely to poor administrative bottlenecks [2]. Funding sources for the NHIS and exempt categories for NHIS premium payment are detailed in the National Health Insurance Authority’s (NHIA) annual reports [3, 4]. The funding sources encompass a 2.5% (value added tax) National Health Insurance Levy (NHIL), return on National Health Insurance Fund Investment; premium fees, donations and contributions to the Social Security and National Insurance Trust.

In terms of service or benefits coverage, the NHIS does not pay for all conditions treated at NHIS-accredited health facilities [2]. Nevertheless, it covers about 95% of the disease burden of Ghana [4]. Yet, cancer and renal (kidney) diseases which could plunge households into catastrophic health expenditures due to the high cost of treatment are not covered by the scheme.

Ghana has experienced a marked improvement in healthcare accessibility with its concomitant improvement in population health outcomes since the introduction of the NHIS in 2003. The literature shows that Ghana’s free maternal care policy has positively impacted antenatal care utilization and supervised delivery [5–7]. In particular, it is evident based on data from selected Districts in the Brong-Ahafo region that facility delivery increased by approximately 20% points between January 2004 and December 2009 largely due to the NHIS subscription in that region [2, 5, 6].

Moreover, Mensah et al. [8] opined that NHIS policy holders who are pregnant are 85.7% more likely to receive prenatal care compared to 72% for non-policy holders. Similarly, 75% of insured expecting mothers deliver at a hospital as compared to 52% for non-insured expecting mothers. Dzakpasu et al. [5] further revealed that NHIS-insured expectant mothers have a greater likelihood of benefiting from postnatal checkups than their non-insured counterparts [2, 5].

Notwithstanding these empirical evidences of the benefits of the NHIS to the people of Ghana, the scheme is confronted with challenges that also threaten its effectiveness and sustainability. Reports of perceived poor quality of services rendered to NHIS subscribers and unwillingness of NHIS card bearers to access health care services with their cards for fear of receiving inferior care [9] are particularly disturbing and demand further investigation using available nationally representative data. Moreover, previous empirical literature alludes to the poor quality of services rendered to NHIS-subscribers as important sustainability threats to the scheme [2, 9–12]. In 2008, a citizen’s assessment of the NHIS by both insured and uninsured clients pointed to perceived poor quality of services provided to insured clients by the NHIS-accredited health facilities in the areas of stock-out of essential drugs and poor attitude of staff as some of the marked quality challenges of the scheme. With reference to stock-out of essential drugs, a staggering 80% of NHIS-insured clients indicated that essential drugs are often not available at the health facility [13].

Indeed, in its 2011 Annual Report, the Ghana Health Service (GHS) [14] concluded that the increased utilization of allopathic healthcare services engendered by the NHIS notwithstanding, its impact on quality healthcare delivery is negligible. The report [14] further stated that on the contrary, the scheme’s introduction had resulted in undue pressure on health infrastructure and staff resulting in longer waiting times and charging of unapproved fees inter alia [14].

It is also evident from the extant literature that patient perceived satisfaction with the quality of healthcare services provided by the scheme has been declining over time. This is especially so in quality indicator areas such as waiting times, preferential treatment for NHIS-policy holders and “quality” of drugs covered by the NHIS [5, 12, 15]. Moreover, clients have also expressed concerns about the delayed issuance of NHIS membership cards and inadequate information on approved benefits package. However, the NHIS has recently sought to address some of these concerns, the most prominent being the introduction of bio-metric membership cards and electronic claims processing to avert the incessant delays in claims reimbursement.

Considering these challenges confronting the NHIS, there is the need to explore, using nationally representative data, the determinants of perceived performance (quality) of the NHIS among rural and urban dwellers. The study focuses on subscribers’ perspectives on the quality of services provided at NHIS offices and NHIS-accredited health care facilities. Understanding these dynamics would help identify and “flag” threats to the NHIS viability. Outcomes of this study are expected to contribute to efforts towards improving the quality of services provided at the levels of the NHIS offices and NHIS-accredited health facilities.

Even though previous scientific endeavors have explored the issue of service quality in the context of the NHIS, many of these efforts in the past have been skewed to only health care facilities without considering the perceived quality of NHIS district and regional offices [16, 17]. Moreover, previous studies on the subject area did not consider the rural-urban dynamics on the perceived service quality [18], a concern which is reinforced by differences in availability of and access to health facilities as well as health workforce in the rural versus urban areas [9]. The literature is replete with studies that examined gender differences in perceived healthcare quality, justifying the need to focus on the gender-perspective as well [19–24]. For instance, using the Consumer Assessment of Healthcare Providers and Systems (CAHPS) Hospital Survey to explore gender differences in inpatient quality care experiences in the USA, Elliot et al. reported that women largely have a less positive experiences with quality of healthcare services than men [23]. The gender differences were more pronounced in the functional quality indicators such as access to information about medicines, discharge information and cleanliness of health facility. Teunissen et al. reported noteworthy gender differences in patients’ quality of care experiences where women were found to have rated hospital quality (consultation privacy) significantly lower (*p* = 0.0001) than men and this was more pronounced in women with higher education [20]. Moreover, a related study revealed that female patients are more likely to express dissatisfaction with nursing care [24].

The closest study in Ghana to our current study is that of Dixon et al. [19]. However, the current study differs fundamentally from that of Dixon et al. [19] on the following reasons;

First, the current study uses the most recent Ghana Demographic and Health Survey (GDHS) data collected in 2014, which is 10 years after the implementation of the NHIS (allowing subscribers ample time to share their experiences with the scheme) for the analysis unlike Dixon et al. that used the 2008 GDHS, just four years after the implementation of the scheme. Second, the current study investigates the phenomenon from a rural-urban perspective while Dixon et al. focused on the gender perspective. Finally, the current study controls for a key variable in whether out-of-pocket payments were incurred by insured clients, an attribute that shapes policy holders’ perception about the quality of NHIS services [25]. It is thus envisaged that the factors that influence the perception of quality by urban subscribers might be distinctly different from that of rural subscribers, an important concern which is nuanced in the literature. Also, this paper is necessitated by the fact that earlier studies had relied largely on primary data limited to particular Districts or Regions [26, 27], thus raising representativeness concerns of such studies.

This paper therefore seeks to address the existing gaps in the literature using the most recent nationally representative Ghana Demographic and Health Survey conducted in 2014. The rural-urban mainstreaming in this study is expected to help policy makers in Ghana, particularly within the National Health Insurance Authority, Ministry of Health, Ghana Health Service, and other relevant stakeholders proffer interventions towards addressing peculiar challenges confronting subscribers of NHIS in Ghana. Findings from this study are expected to help inform evidence-based policy decisions towards promoting its sustainability and ultimately improve universal health coverage, population health and wellbeing.

## Methods

### Data

The study is based on data from the internationally recognized Demographic and Health Surveys (DHSs) Programme. This project conducts countrywide population and health surveys across the globe. Since its inception, six rounds of the Ghana Demographic and Health Surveys (GDHSs) have been conducted (1988, 1993, 1998, 2003, 2008 and 2014) by the Ghana Statistical Service (GSS), Ghana Health Service (GHS), the National Public Health Reference Laboratory (NPHRL) and ICF International. The study utilized data from the men’s and women’s questionnaires of the 2014 GDHS. The 2014 GDHS is the most recent of such nationally representative surveys. It successfully interviewed 4388 (2050 urban and 2338 rural) men aged 15–59 and 9396 (4602 urban and 4794 rural) women aged 15–49 from 11,835 (5939 urban and 5896 rural) households. The respective response rates for the successfully interviewed men, women, and households are approximately 95% (94% urban and 97% rural), 97% (97% urban and 98% rural), and 99% (98% urban and 99% rural) [28].

The GDHSs have been widely used in many quantitative studies mainly due to their provision of reliable and quality information [28]. They follow a two-stage sampling design. The first stage involves the selection process where sample points (clusters) which consist of enumeration areas (EAs) are randomly selected. The 2014 GDHS selected 427 clusters in total. This consisted of 216 and 211 clusters in urban and rural areas respectively. Households are systematically sampled in the second stage of the GDHSs; approximately 30 households from each cluster were selected in the 2014 GDHS. This resulted in a total of 12,831 (6492 urban and 6339 rural) households selected. According to GSS, GHS, and ICF International [28], because Ghana’s administrative regions had an almost equal sample size in the 2014 GDHS, though weighting factors have been added to the result in the dataset being proportional at the countrywide level, the sample is not self-weighted at that level.

The study utilized data on 10,065 (4992 urban and 5073 rural) men and women out of the total 13,784 persons successfully sampled. This is because these were the individuals who had registered for the scheme and had observations for all the variables of interest.

### Explained variable

A component on Ghana’s National Health Insurance Scheme (NHIS) was introduced in the recent surveys. Owners of NHIS cards were asked to indicate whether they get worse, same, or better quality of services than non-card holders. The response to this question was used as the outcome variable of the study. Table 1 presents a description and measurement of the outcome variable.

**Table 1:** Description and measurements of outcome variable

Outcome variable	Description and measurement
Perceived quality	Quality perception of NHIS services by card holders: worse = 0+; same = 1; better = 2;

*+*implies reference category

### Explanatory variables

The dataset contains information on the Socioeconomic and Demographic (SED) characteristics of individuals interviewed. These SED characteristics include education, gender, age, region of residence, wealth status, among others. Description and measurement of the selected explanatory variables are presented in Table 2. These variables were selected with recourse to existing literature.

**Table 2:** Description and measurements of explanatory variables

Explanatory variables	Description
Age	Age of respondent (years)
Age square	Age of respondent square: square of age
Out-of-pocket payment	Out-of-pocket (OoP) healthcare payment status: doesn’t pay = 0+; pays = 1
Wealth	Wealth status of respondent^a^: poor = 0+; average = 1; rich = 2
Media access	Access to media: none = 0+; partial access (used at least newspapers, radio, or tv) = 1; full access (used newspapers, radio, and tv) = 2
Education	Educational status of respondent: no education = 0+; basic = 1; at least secondary = 2
Region	Administrative region of residence: Greater Accra = 0+; Western = 1; Central = 2; Volta = 3; Eastern = 4; Ashanti = 5; Brong-Ahafo = 6; Northern = 7; Upper East = 8; Upper West = 9
Male	Gender of respondent: male = 1; female =0+
Urban	Place of residence: urban = 1; rural = 0+

*+*implies reference category

^a^Computed by recoding the original wealth quintile in the GDHS; poorest/poorer = poor, middle = average, and richer/richest = rich

### Estimation technique

The outcome variable, perceived quality of services - better, same or worse, exhibits natural ordering such that better services are preferred to same services, and same are preferred to worse services. An ordered logistic regression model was utilized to investigate policy holders’ perceptions of quality of service provided to rural and urban subscribers of Ghana’s NHIS. The discrete dependent variable, y, takes on arbitrary but ascending values of 0, 1, and 2 for the categories worse, same, and better respectively. The ordered logistic regression model is expressed as;

$$ {y}_i={x}_i\beta +{\varepsilon}_i $$

where *y*_*i*_ is the ranked dependent variable; *x*_*i*_ denotes the vector of independent variables; and *β* represents the vector of coefficients; and *ε*_*i*_ being the error term.

We provide joint and separate estimations for rural and urban subsamples. Also, further analyses were performed to explore the correlates of perceived quality of services provided to men and women in rural and urban areas. Consequently seven empirical models would be estimated. These models are specified as follows;

1 $$ \boldsymbol{Full}\ \boldsymbol{sample}:{Perceived\ quality}_i={\beta}_0+{\beta}_1{Age}_i+{\beta}_2 Age\ {squared}_i+{\beta}_3 OoP\ {payment}_i+{\beta}_4{Wealth}_i+{\beta}_5{Media\ access}_i+{\beta}_6{Education}_i+{\beta}_7{Region}_i+{\beta}_8{Male}_i+{\beta}_9{Urban}_i+{\varepsilon}_i $$

2 $$ \boldsymbol{Urban}\ \boldsymbol{subsample}:{Perceived\ quality}_{ij}={\beta}_0+{\beta}_1{Age}_i+{\beta}_2 Age\ {squared}_i+{\beta}_3 OoP\ {payment}_i+{\beta}_4{Wealth}_i+{\beta}_5{Media\ access}_i+{\beta}_6{Education}_i+{\beta}_7{Region}_i+{\beta}_8{Male}_i+{\varepsilon}_i $$

where: j = {0(both); 1(male); 2(female)}; i = respondent; M_i_ was excluded when j = 1 or j = 2.

3 $$ \boldsymbol{Rural}\ \boldsymbol{subsample}:{Perceived\ quality}_{ij}={\beta}_0+{\beta}_1{Age}_i+{\beta}_2 Age\ {squared}_i+{\beta}_3 OoP\ {payment}_i+{\beta}_4{Wealth}_i+{\beta}_5{Media\ access}_i+{\beta}_6{Education}_i+{\beta}_7{Region}_i+{\beta}_8{Male}_i+{\varepsilon}_i $$

where: j = {0(both); 1(male); 2(female)}; i = respondent; M_i_ was excluded when j = 1 or j = 2.

The empirical models were estimated using maximum-likelihood (MLE) technique in STATA 15.0. Survey design was considered in all the estimations.

## Results

### Summary statistics

Table 3 presents summary statistics of the variables used in the study. It suggests that subscribers in rural Ghana on average have better perception of the quality of services provided by the NHIS than subscribers in urban Ghana. For instance, 39 and 27% of urban subscribers compared with 28 and 41% of rural subscribers perceived the quality of NHIS services as worse and better respectively. On average, there are more males (28%), poor individuals (61%), persons who partially use media (79%) or do not use media at all (8%), and persons with basic education (20%) or no education (24%), subscribers who do not incur additional out-of-pocket health expenses (62%), in the rural subsample than in the urban subsamples. Independent chi-square statistics were performed to test for significant differences in the proportion of the characteristics of the subscribers in the two sub-samples (rural and urban).

**Table 3:** Summary statistics of variables (rural vs. urban)

Explanatory variables	Full Sample (*n* = 10,065)		Urban Subsample (*n* = 4992)		Rural Subsample (*n* = 5073)			
	Mean	SE	Mean	SE	Mean	SE	F-statistic	*P*-value
Age	30.77	0.14	30.84	0.20	30.69	0.18	–	–
Perceived quality							27.91	0.00
Worse	0.34	0.01	0.39	0.02	0.28	0.01		
Same	0.33	0.01	0.34	0.01	0.31	0.01		
Better	0.34	0.01	0.27	0.02	0.41	0.02		
Out-of-pocket payment status							10.90	0.00
Doesn’t pay+	0.58	0.01	0.55	0.01	0.62	0.02		
Pays	0.42	0.01	0.45	0.01	0.38	0.02		
Wealth status							187.81	0.00
Poor+	0.33	0.02	0.08	0.01	0.61	0.03		
Average	0.20	0.01	0.16	0.01	0.25	0.02		
Rich	0.47	0.02	0.76	0.02	0.14	0.02		
Access to Media							63.38	0.00
None+	0.05	0.00	0.03	0.00	0.08	0.01		
Partial	0.72	0.01	0.66	0.01	0.79	0.01		
Full	0.22	0.01	0.31	0.01	0.12	(0.01		
Educational status							60.07	0.00
No education+	0.16	0.01	0.09	0.01	0.24	0.02		
Basic	0.15	0.01	0.11	0.01	0.20	0.01		
At least secondary	0.69	0.01	0.80	0.01	0.56	0.02		
Region							6.10	0.00
Greater Accra+	0.16	0.02	0.27	0.04	0.04	0.02		
Western	0.12	0.02	0.10	0.02	0.14	0.03		
Central	0.08	0.02	0.06	0.01	0.11	0.04		
Volta	0.08	0.01	0.06	0.02	0.11	0.02		
Eastern	0.10	0.02	0.09	0.02	0.11	0.02		
Ashanti	0.20	0.03	0.24	0.04	0.16	0.04		
Brong-Ahafo	0.10	0.01	0.09	0.02	0.10	0.02		
Northern	0.09	0.02	0.05	0.01	0.13	(0.03		
Upper East	0.04	0.01	0.02	0.01	0.07	0.02		
Upper West	0.02	0.00	0.01	0.00	0.04	0.01		
Gender							5.24	0.02
Female+	0.74	0.00	0.75	0.01	0.72	0.01		
Male	0.26	0.00	0.25	0.01	0.28	0.01		

All, but age, are in column proportions. Survey design accounted for in estimations. SE denotes linearized standard errors. + denotes reference category. ++ Media use in this study means access to information on the NHIS (i.e. radio, TV etc.). *** p < 0.01, ** p < 0.05. Source: Computed by authors from 2014 GDHS

Results indicate that subscribers in the rural subsample are significantly different from subscribers in the urban sub-sample in terms of the selected socioeconomic and demographic characteristics. This observed heterogeneity could result in different factors affecting the quality perception of services provided by Ghana’s NHIS in rural and urban areas. This justifies our disaggregation of the full sample into rural and urban sub-samples to verify whether there are any differences between the urban and rural policy holders’ perception of service quality.

### Multivariate analysis

To determine factors associated with subscribers’ perception of NHIS service quality, ordered logistic regression (OLR) was conducted (see Table 4). For effective policy intervention, besides the full sample estimation, we attempt disaggregating the full sample across space (rural versus urban) and further across gender (male versus females). The gender disaggregation is performed across the respective rural and urban subsamples to observe probable differences in the determinants across gender in these localities. Consequently, seven (7) separate results are reported in Table 4. It is observed that at all the levels of the analysis, some disparities exist. These disparities are noted to be more distinct in the male-female disaggregation of the rural and urban sub-samples. Generally, the following are found to be the most important predictors of perceived quality of services provided to subscribers of the NHIS in Ghana (see Table 4, level 1): age, out-of-pocket healthcare payment status, wealth status, and administrative region of residence.

**Table 4:** Odds ratios from ordered logistic regression estimation of the correlates of perceived quality of services in Ghana, 2014

Explanatory variables	Full sample	Urban subsample			Rural subsample		
	(level 1) all OR(95% CI)	(level 2) All OR (95% CI)	(level 3) male OR (95% CI)	(level 4) female OR 95% CI)	(level 5) all OR 95% CI)	(level 6) male OR (95% CI)	(level 7) female OR (95% CI)
Age	0.95***	0.94***	0.94	0.90***	0.97	0.90***	0.97
	(0.92–0.98)	(0.90–0.97)	(0.89–1.01)	(0.85–0.95)	(0.93–1.00)	(0.85–0.96)	(0.93–1.02)
Age square	1.00***	1.00***	1.00	1.00***	1.00	1.00***	1.00
	(1.00–1.00)	(1.00–1.00)	(1.00–1.00)	(1.00–1.00)	(1.00–1.00)	(1.00–1.00)	(1.00–1.00)
Out-of-pocket payment status							
Doesn’t pay+ 1.00	1.00		1.00	1.00	1.00	1.00	1.00
Pays	0.85**	0.96	1.09	0.90	0.74***	0.93	0.64***
	(0.76–0.96)	(0.81–1.13)	(0.76–1.55)	(0.75–1.09)	(0.62–0.89)	(0.70–1.25)	(0.51–0.80)
Wealth status							
Poor+	1.00	1.00	1.00	1.00	1.00	1.00	1.00
Average	0.76***	0.90	0.84	0.93	0.70***	0.86	0.65***
	(0.65–0.89)	(0.71–1.15)	(0.50–1.38)	(0.69–1.25)	(0.59–0.84)	(0.59–1.28)	(0.51–0.82)
Rich	0.68***	0.80	1.04	0.75	0.70**	0.41***	0.83
	(0.55–0.84)	(0.61–1.05)	(0.63–1.71)	(0.54–1.03)	(0.50–0.97)	(0.23–0.74)	(0.55–1.27)
Access to Media							
None+ 1.00		1.00	1.00	1.00	1.00	1.00	1.00
Partial	0.92	1.05	1.68	1.27	0.86	0.59	0.83
	(0.77–1.12)	(0.72–1.52)	(0.92–3.09)	(0.83–1.94)	(0.69–1.07)	(0.26–1.36)	(0.63–1.09)
Full	0.88	1.07	2.02**	1.26	0.69**	0.58	0.52***
	(0.71–1.10)	(0.73–1.59)	(1.05–3.89)	(0.81–1.97)	(0.50–0.95)	(0.22–1.49)	(0.35–0.77)
Educational status							
No education+	1.00	1.00	1.00	1.00	1.00	1.00	1.00
Basic	0.97	1.02	1.17	0.94	0.92	1.18	0.89
	(0.82–1.15)	(0.78–1.35)	(0.46–3.00)	(0.72–1.25)	(0.74–1.14)	(0.72–1.94)	(0.68–1.17)
At least secondary	0.89	0.83	0.59	0.84	0.96	1.03	1.05
	(0.76–1.03)	(0.65–1.05)	(0.26–1.33)	(0.65–1.08)	(0.80–1.16)	(0.66–1.62)	(0.81–1.37)
Region							
Greater Accra+	1.00	1.00	1.00	1.00	1.00	1.00	1.00
Western	2.58***	2.46***	1.92***	2.78***	3.28***	0.60	6.26***
	(2.02–3.29)	(1.85–3.26)	(1.17–3.13)	(1.90–4.07)	(2.18–4.95)	(0.21–1.71)	(3.73–10.48)
Central	2.46***	2.60***	7.70***	1.92***	2.92***	8.95***	2.25***
	(1.89–3.21)	(1.84–3.67)	(2.97–19.96)	(1.28–2.89)	(1.96–4.35)	(2.53–31.63)	(1.46–3.48)
Volta	1.07	1.20	2.87***	0.94	1.27	1.75	1.13
	(0.79–1.46)	(0.74–1.94)	(1.44–5.73)	(0.52–1.68)	(0.84–1.93)	(0.64–4.77)	(0.68–1.86)
Eastern	2.10***	2.11***	2.42***	2.15**	2.63***	1.95	3.14***
	(1.47–3.00)	(1.30–3.43)	(1.40–4.19)	(1.15–4.04)	(1.56–4.44)	(0.70–5.44)	(1.63–6.05)
Ashanti	0.85	0.73	3.15***	0.44***	1.39	2.45	1.06
	(0.65–1.11)	(0.52–1.01)	(1.74–5.71)	(0.29–0.67)	(0.92–2.08)	(0.85–7.06)	(0.63–1.78)
Brong-Ahafo	0.98	1.13	1.57	1.04	1.08	0.84	1.22
	(0.76–1.26)	(0.84–1.53)	(0.85–2.92)	(0.71–1.52)	(0.72–1.64)	(0.30–2.36)	(0.70–2.12)
Northern	1.83***	2.45***	5.06***	1.95***	2.00***	2.26	2.01***
	(1.37–2.46)	(1.76–3.42)	(2.31–11.10)	(1.30–2.92)	(1.30–3.07)	(0.58–8.83)	(1.21–3.33)
Upper East	2.15***	2.12***	5.47***	1.56	2.69***	6.33***	2.10***
	(1.59–2.90)	(1.38–3.27)	(3.02–9.89)	(0.94–2.58)	(1.76–4.11)	(1.96–20.39)	(1.29–3.42)
Upper West	1.83***	2.65***	6.34***	2.01**	1.89**	2.90	1.60
	(1.25–2.67)	(1.72–4.08)	(3.12–12.90)	(1.12–3.60)	(1.11–3.24)	(0.94–8.92)	(0.81–3.19)
Gender							
Female+	1.00	1.00			1.00		
Male	0.98	1.06			0.92		
	(0.79–1.20)	(0.81–1.39)			(0.65–1.28)		
Place of residence							
Rural+	1.00						
Urban	0.89						
	(0.75–1.06)						
Constant cut1	0.17***	0.19***	0.55	0.10***	0.25***	0.09***	0.26***
	(0.10–0.28)	(0.09–0.40)	(0.14–2.27)	(0.04–0.24)	(0.12–0.52)	(0.02–0.46)	(0.11–0.62)
Constant cut2	0.73	0.92	2.89	0.50	1.01	0.32	1.21
	(0.45–1.20)	(0.46–1.85)	(0.70–11.82)	(0.22–1.15)	(0.49–2.07)	(0.06–1.68)	(0.52–2.80)
Observations	10,065	4992	1302	3690	5073	1420	3653
F-statistic	14.48 (0.00)	8.06 (0.00)	4.36 (0.00)	6.29 (0.00)	8.36 (0.00)	5.60 (0.00)	8.07 (0.00)

Survey design accounted for in estimations. *P*-values in parentheses for the F-statistic. OR denotes odds ratio; CI denotes confidence interval. + denotes reference category (OR = 1.00). ++Media use in this study means access to information on the NHIS (i.e. radio, TV etc.). *** p < 0.01, ** p < 0.05. Source: Computed by authors from 2014 GDHS

Though insignificant, the results from the full sample give an indication that compared to rural subscribers, urban subscribers are less likely to perceive the quality of services provided by the NHIS as same or better than worse (see Table 4, level 1). Age is found to be significant in the full sample, urban subsample (see Table 4, level 2) and not in the rural subsample (see Table 4, level 5). While younger urban subscribers have worse than same or better perception about the quality of services provided, their older counterparts have higher likelihood of perceiving the services provided by the NHIS as same or better than worse (see Table 4, level 2). Also, rural subscribers who still pay out-of-pocket at health facilities are found to be less likely to perceive the quality of services provided as same or better than worse. Compared to rural subscribers who are least wealthy, those with average and greater wealth are found to have a lower likelihood to perceive the quality of services provided by the NHIS as better or same than worse (see Table 4, level 5). The results in Table 4, level 5 show that while rural subscribers who had access to newspapers, T.V., and radio are significantly less likely to have same or better than worse opinions of the quality of services provided by the NHIS, access to all these forms of media proved to have no significant effect on the perceived quality of service among urban subscribers. Apart from these observed differences, region of residence is a significant predictor of perceived quality of NHIS services in the full (Table 4, level 1) sample and among both rural and urban subscribers. For instance, in the full sample (level 1), it is revealed that compared to the Greater Accra Region (reference region), residents of Western Region, Central Region, Eastern Region, Upper East and Upper West Region are 2.58, 2.46, 2.10, 2.15 and 1.83 times more likely to perceive NHIS services rendered to subscribers as the same or better relative to perceiving the services as worse. The positive perception of NHIS services as better or same compared to worse is consistent with the rural and urban and gender sub-samples. Of particular interest is the highly significant and positive effect of the rural female (level 7) and rural male residents (level 6) of the Western region and Upper East Region respectively on perceived quality of care. The results indicate that rural female residents in the Western Region and rural male residents in Upper East Regions are 6.26 and 6.33 times more likely to have a positive perception of NHIS services as better or same compared to worse respectively.

 Level 3 and level 6 of Table 4 reveal that apart from region of residence which is positive and significant among both rural and urban male subscribers, age and wealth status are significant among rural male subscribers only. Furthermore, in addition to region of residence which is a significant predictor among female subscribers in both rural and urban areas, age is significant among female subscribers in urban areas only, while out-of-pocket health payment status, wealth status, access to media are significant among females in rural areas only (see, level 4 and level 7 of Table 4) emphasizing the peculiarity of the correlates of perceived NHIS quality by gender and location. Thus far, none of the previous and related studies [17, 18, 21] had controlled for out-of-pocket payment on perceived quality of NHIS services. Findings from this study reveal that incurring out-of-pocket expenditures are associated with a lower perception of quality of NHIS services. Specifically, subscribers who made out-of-pocket expenditures were 0.85, 0.74 and 0.64 times less likely to perceive the quality of NHIS services as the same or better as compared to worse in the full sample (level 1), rural full sample (level 5) and rural female sample (Level 7) respectively.

## Discussion

The findings from the ordered logistic regression support the conclusions drawn from the chi-square statistics that different factors affect the perception of quality of NHIS services provided to urban and rural subscribers.

Consistently, the study links perceiving the quality of services provided by Ghana’s NHIS as same as or better than worse to being deprived. That is, poor subscribers are more likely to have positive opinions about the quality of services. This is based on the findings that wealthier subscribers as well as subscribers who reside in urban areas and Greater Accra region (reference region) of Ghana are less likely to have better opinions about the quality of services. According to the Ghana Statistical Service [28], 78 % of the poor in Ghana live in rural areas. Also, majority of persons living in extreme poverty in Ghana are found in the three northern regions [29, 30]. These evidences support the positive association between poverty and having better perceptions of the quality of services provided by Ghana’s NHIS. The positive association between deprivity or poverty (wealth status) and perceived quality of NHIS services may be partly explained by the fact that the poor live mostly in communities that lack adequate access to health facilities and health personnel. Given the limited choice of health facilitties and qualified health personnel, subscribers tend to be content with the quality of NHIS services provided in such communies and the reverse is true for the relatively rich urban dwellers where both private and public health facilities and health perosnnel are relatively abundant.

Dixon et al. [18] made similar findings based on evidence from the 2008 GDHS report. The findings from the current study which is drawn from the 2014 GDHS reveal that poverty status remains the most important factor in determining how Ghanaians perceive the quality of services provided by the NHIS. Several plausible reasons were identified by Dixon et al.. [19] to account for this situation. Most striking of them is the fact that wealthier households have access to services provided by other forms of insurance and healthcare payment systems both within and outside this country, they are more likely to compare the services rendered by the NHIS to such, and hence rate the quality of services of the NHIS as worse.

Adding to existing knowledge, this study reveals that while wealth status is neither significant among female nor male subscribers in urban areas, it is significant among both female and male subscribers in rural areas. The significance of wealth status among female subscribers contradicts Dixon et al. [19] probably due to the fact that the current study uses a more recent data (2014) relative to the 2008 data utilized by Dixon et al. [19], although their study also employed a nationally represenatiave data. Futhermore, we observe that there is a differential wealth effect on gender with regard to perceived quality of NHIS services. While males in the ‘rich’ wealth index in rural areas are significantly associated with perceived quality of NHIS, for females, it is rather the average wealth index that proved significant. Affirming the argument put across by Dixon et al.. [19], the above observation further expatiates the gender dynamics of intra-household resource allocation. Though age of the subscriber proved significant among female subscribers in urban areas, it was found to be significant among male subscribers in rural areas. Both findings show that subscribers are less likely to perceive the quality of services provided by the NHIS as same as or better than worse per advancement in age. The significance of the age square variable is that it captures non-linear effect of age on perceived quality. Our results however reveals that older subscribers are more likely to be identified with same or better than worse opinions about the quality of services derived by the NHIS. This could plausibly be due to the ‘respect’ and preferential treatment given to elderly people in Ghana. Besides, plausibly due to the fact that women in rural Ghana are among the most deprived, the study finds an adverse effect of their having to pay out-of-pocket on health services on their perception of the quality of services provided by the scheme. Moreover, while male subscribers who have full access to media in urban areas are found to be more likely to perceive the quality of services provided by the NHIS as same as or better than worse, female subscribers who have full access in rural areas are less likely to perceive the quality of services as same or better than worse.

One of the stimulating findings from this study is the significant regional differences in perceived quality of healthcare across gender and location. For instance, in the full sample (level 1), the Western region was associated with the highest likelihood of perceived quality of healthcare (OR = 2.58) as compared to the Greater Region (reference region). Andoh-Adjei et al. [31] in a recent study evaluated the perceived quality of healthcare delivery under the capitation payment system in Ghana and found that significant regional differences exist at least for the three regions controlled for in their study. Particularly, the study found that respondents in the Central Region had a higher perception of health quality than the respondents from the Ashanti Region. Results from our study is consistent with Andoh-Adjei et al. [31] as shown by the higher likelihood of perceived quality of healthcare in the Central Region (OR = 2.46; *p* = 0.001) as compared to the Ashanti Region (OR = 0.85, *p* = 0.1). In fact, the dummy for Ashanti Region proved insignificant in all the estimations, whether by gender or location (See Table 4 levels 1 through 7)). The low perceived quality of healthcare in the Ashanti Region has been blamed on a number of factors including the introduction of the capitation grant on pilot basis between 2012 and 2016. Unsurprisingly, lack of proper appreciation of the capitation system of payment by some providers and clients coupled with subtle politicization by some politicians might have created a wrong impression about the quality of healthcare delivered at NHIS-accredited facilities in the Ashanti Region [31]. With regard to gender however, male residents in the Central Region had the highest odds (OR = 7.70) of perceiving quality of healthcare as same or better when place of residence (rural vs urban) is controlled for, followed by the Upper West Region (OR = 6.34) which paradoxically is the region with the least health facilities and number of health personnel in the country. The higher perception of quality of healthcare in the deprived Upper West Region could be attributable to the lack of alternative health facilities in the region since most of the districts have either one public and/or mission facilities unlike the Greater Accra region which boasts of the highest number of private health facilities. It can also be argued that the relatively high perceived quality of healthcare in the Upper West region for instance, could be explained by the complementary role of health-related NGOs in the region.

Finally, while the study produces interesting results regarding the correlates of perceived quality of services rendered by the NHIS, it is admittedly fraught with certain limitations. More pronouncedly, the measurement of quality is based on a subscriber’s experience with the scheme and not on scientific evaluation. Such self-evaluation of quality of healthcare services suffers from biasedness especially when subscribers are empathized with or treated in a more professional manner or better still receive services from familiar providers. Moreover, since this is a cross-sectional study, we can only infer associations and not causations. The results from this study should thus be interpreted with these limitations in mind. Finally, given the fact that wealth status proved significant among both female and male subscribers in rural but not in urban areas, it is suggestive that the computation of the wealth index ought to be sensitive to the residential (rural vs urban) status of respondents. Nevertheless, the current calculation of the wealth index in the GDHS does not account for the rural-urban differences in wealth, i.e., there are no separate estimates of the wealth index for rural and urban areas. Perhaps, our findings might have been more revealing if the data permitted such disaggregation. We therefore admit this as one of the weaknesses of this paper. However, the limitations acknowledged in this paper by no means undermine the validity of our findings.

## Conclusion

Previous studies on the rural-urban dynamics of perceived quality of services provided to subscribers of Ghana’s NHIS have been limited. This current study used the most recent nationally representative Ghana Demographic and Health Survey [28] to analyze NHIS-subscribers’ perceptions of the quality of service. The paper explored the experiences of the NHIS from the perspective of policy holders in rural and urban contexts.

Rural subscribers of the NHIS were found to identify more with better perception of quality of services provided by the NHIS than urban subscribers. Findings from the chi-square statistics further indicated that rural subscribers are significantly different from urban subscribers in terms of the selected socioeconomic and demographic characteristics. Age, out-of-pocket healthcare payment status, region of residence, wealth status, and access to media were found to be significant predictors of perceived quality of services provided to rural and urban subscribers of the NHIS. Significance of these variables however varied among men and women in rural and urban areas.

The study concludes that different factors affect the perception of quality of services provided by Ghana’s NHIS between rural and urban subscribers. It therefore recommends that public health policies geared toward improvement in NHIS-related services should use different strategies in the rural and urban areas. Given the consistently negative association between out-of-pocket payments and perceived quality of care (which is more prevalent among females in rural areas), the scheme should strive to reduce such expenditures which could plunge subscribers into catastrophic expenditures, by timely reimbursing NHIS-accredited facilities to avert a situation where delayed reimbursement forces providers to charge fees. Also, where certain services are not covered by the scheme, the NHIA should embark on educational campaigns to educate subscribers on the approved benefits package for policy holders, otherwise such subscribers will inevitably have a negative perception about the quality of NHIS services. The positive association between access to full media (newspaper, radio and TV) and perceived quality of NHIS also makes a strong case for promoting access to media especially among women in the rural areas to improve their knowledge on health-related issues. The proliferation of private radio stations that feature health education in rural communities is a commendable initiative that should be scaled-up to promote particularly maternal child care.

## Abbreviations

DHS: Demographic and Health Survey
GDHS: Ghana Demographic and Health Survey
GSS: Ghana Statistical Service
NHIF: National Health Insurance Fund
NHIS: National Health Insurance Scheme
NPHRL: National Public Health Reference Laboratory
OLR: Ordered Logistic Regression
SED: Socioeconomic and Demographic

## References

[1] Government of Ghana. The National Health Insurance Act: Act 852. Accra: Ministry of Health; 2012.

[2] Alhassan R, Spieker N, Nketiah-Amponsah E, Arhinful D, Rinke de Wit T (2016). Assessing the impact of community engagement interventions on health worker motivation and experiences with clients in primary health facilities in Ghana: a randomized cluster trial. PLoS One.

[3] National Health Insurance Authority (NHIA) (2013). Annual report.

[4] National Health Insurance Authority (NHIA) (2012). Annual report.

[5] Dzakpasu S, Soremekun S, Manu A, ten Asbroek G, Tawiah C, Hurt L (2012). Impact of delivery care on health facility delivery and insurance coverage in Ghana’s Brong-Ahafo region. PLoS One.

[6] Blanchet N, Fink G, Osei-Akoto I (2012). The effect if Ghana’s National Health Insurance Scheme on health care utilization. Ghana Med J.

[7] Mensah J, Oppong J, Bobi-Barimah K, Frempong G, Sabi W (2010). An evaluation of the Ghana national health insurance scheme in the context of the health MDGs Working Paper No 40.

[8] Witter S, Adjei S, Armar-Klemesu M, Graham W (2009). Providing free maternal health care: ten lessons from an evaluation of the national delivery exemption policy in Ghana. Glob Health Action.

[9] Alhassan R, Duku S, Jassens W, Nketiah-Amponsah E, Spieker N, van Ostenberg P (2015). Comparison of perceived and technical health care quality in primary health facilities: implications for a sustainable national health insurance scheme in Ghana. PLoS One.

[10] Abiiro G, McIntyre D (2012). Universal financial protection through National Health Insurance: a stakeholder analysis of the proposed one-time premium payment policy in Ghana. Health Policy Plan.

[11] Adei D, Kwadwo O, Diko V, An S (2009). Assessment of the Kwabre District mutual health insurance scheme in Ghana. Curr Res J Soc Sci.

[12] Fusheini D, Marnoch G, Grey A (2012). The implementation of the National Health Insurance Programmeme in Ghana±an institutional approach.

[13] National Development Planning Commission (NDPC). Citizens’ assessment of the National Health Insurance Scheme of Ghana, towards a sustainable health care financing arrangement that protects the poor. Accra: National Development Planning Commission; 2009.

[14] Ghana Health Service (GHS) (2011). Annual Report.

[15] Ministry of Health (MoH). Independent review of 2006 programmeme of work (POW). First Draft. Accra: Ministry of Health; 2007.

[16] Alhassan R, Nketiah-Amponsah E, Akazili J, Spieker N, Arhinful D, Rinke de Wit T (2016). Efficiency of private and public primary health facilities accredited by the National Health Insurance Authority in Ghana. BMC Cost Eff Resour Alloc.

[17] Alhassan R, Spieker N, Nketiah-Amponsah E, Arhinful D, Rinke de Wit T (2016). Perspectives of frontline health workers on Ghana's National Health Insurance Scheme before and after community engagement interventions. BMC Health Serv Res.

[18] Nketiah-Amponsah E, Hiemenz U (2009). Determinants of consumer satisfaction of health Care in Ghana: does choice of health care provider matter?. Global J Health Sci.

[19] Dixon J, Tenkorang EY, Luginaah I (2013). Ghana’s National Health Insurance Scheme: a National Level Investigation of members’ perceptions of service provision. BMC Int Health Hum Rights.

[20] Teunissen TAM, Rotink ME, Lagro-Janssen ALM (2016). Gender differences in quality of care experiences during hospital stay: a contribution to patient-centered healthcare for both men and women. Patient Educ Couns.

[21] Cleary PD, Zaslavsky AM, Cioffi M (2000). Sex differences in assessment of the quality of Medicare managed care. Womens Health Issues.

[22] Sofaer S, Firminger K (2005). Patient perception of the quality of health services. Annu Rev Public Health.

[23] Elliott MN, Lehrman WG, Beckett MK, Goldstein E, Hambarsoomian K, Giordano LA (2012). Gender differences in patients’ perceptions of inpatient care. Health Serv Res.

[24] Foss C (2002). Gender Bias in nursing care? Gender-related differences in patient satisfaction with the quality of nursing care. Scand J Caring Sci.

[25] Brugiavini A, Pace N (2016). Extending health insurance in Ghana: effect of the national health insurance scheme on maternity care. Heal Econ Rev.

[26] Duku SKO, Nketiah-Amponsah E, Janssens W, Pradhan M (2018). Perceptions of healthcare quality in Ghana: does health insurance status matter?. PLoS One.

[27] Badu E, Agyei-Baffour P, Acheampong IO, Opoku MP, Addei-Donkor K. Perceived satisfaction with health services under national health insurance scheme; clients’ perspectives. In J Health Plann Manage. 2018. 10.1002/hpm.2711. 10.1002/hpm.2711 30468521

[28] Ghana Statistical Service (GSS) (2015). Ghana Health Service (GHS), & ICF International. Ghana Demographic and Health Survey 2014.

[29] Ghana Statistical Service (2007). Pattern and Trends of Poverty in Ghana 1991–2006.

[30] Ghana Statistical Service (2014). Ghana Living Standards Survey, Report of Sixth Round (GLSS6).

[31] Andoh-Adjei FX, Nsiah-Boateng E, Asante FA, Spaan E, der Velden KV (2018). Perception of quality of health care delivery under capitation payment: a cross-sectional survey of health insurance subscribers and providers in Ghana. BMC Fam Pract.

